# Mesenchymal stem cells ameliorate rhabdomyolysis-induced acute kidney injury via the activation of M2 macrophages

**DOI:** 10.1186/scrt469

**Published:** 2014-06-24

**Authors:** Yanqiu Geng, Li Zhang, Bo Fu, Jianrong Zhang, Quan Hong, Jie Hu, Diangeng Li, Congjuan Luo, Shaoyuan Cui, Fei Zhu, Xiangmei Chen

**Affiliations:** 1 Department of Nephrology, Chinese PLA General Hospital, Chinese PLA Institute of Nephrology, State Key Laboratory of Kidney Diseases, National Clinical Research Center of Kidney Diseases, Beijing, China; 2 Department of Nephrology, General Hospital of Chinese People’s Armed Police Forces, Beijing, China

## Abstract

**Introduction:**

The mortality of rhabdomyolysis-induced acute kidney injury (AKI) is still high, as there is no effective therapy. It has been shown that bone marrow-derived mesenchymal stem cells (MSCs) can induce M2 macrophages, which mediate MSC protection in other experimental inflammation-related organ injury. This study was designed to investigate the protective effects of macrophage activation in MSC therapy of rhabdomyolysis-induced AKI.

**Methods:**

MSCs were injected into glycerol-induced rhabdomyolysis mice. Renal injury was evaluated using the serum creatinine, urea nitrogen, renal pathology and acute tubular necrosis score. The distribution of MSCs was detected using two-photon fluorescence confocal imaging. Immunofluorescence of anti-F4/80 and anti-CD206 was performed to determine macrophages and M2 macrophages in the tissues of the kidney, and M2 macrophage infiltration was also evaluated using western blotting analyses. After depletion of macrophages using clodronate liposomes at the phase of kidney repair, renal injury was re-evaluated. RAW 264.7 macrophages were incubated with lipopolysaccharide and co-cultured with MSCs and subsequently visualised using immunofluorescence staining and flow cytometry analysis. Finally, disparate phenotype macrophages, including normal macrophages (M0), lipopolysaccharide-stimulated macrophages (M1), and MSC-co-cultured macrophages (M2), were infused into mice with AKI, which were pre-treated with liposomal clodronate.

**Results:**

*In vivo* infusion of MSCs protected AKI mice from renal function impairment and severe tubular injury, which was accompanied by a time-dependent increase in CD206-positive M2 macrophage infiltration. In addition, depleting macrophages with clodronate delayed restoration of AKI. *In vitro*, macrophages co-cultured with MSCs acquired an anti-inflammatory M2 phenotype, which was characterised by an increased expression of CD206 and the secretory cytokine interleukin (IL)-10. The concentrations of IL-10, IL-6 and tumor necrosis factor α were evaluated using enzyme-linked immunosorbent assay. Furthermore, macrophage-depleted mice with intramuscular injection of glycerol were subjected to a single injection of different types of RAW 264.7 macrophages. Mice infused with M0 and M1 macrophages suffered a more severe histological and functional injury, while mice transfused with MSC-educated M2 macrophages showed reduced kidney injury.

**Conclusions:**

Our findings suggested that MSCs can ameliorate rhabdomyolysis-induced AKI via the activation of macrophages to a trophic M2 phenotype, which supports the transition from tubule injury to tubule repair.

## Introduction

Rhabdomyolysis is a syndrome involving the damage and breakdown of skeletal muscle, causing myoglobin and other intracellular proteins and electrolytes to leak into the circulation [1]. Acute kidney injury (AKI) is a serious complication of severe rhabdomyolysis, and the prognosis is substantially worse if renal failure develops [2]. Because there is no effective therapy for AKI, the mortality associated with rhabdomyolysis-induced AKI is considerably high, particularly in crush syndrome. Our previous research showed that the mortality of victims with rhabdomyolysis-related AKI from the Wenchuan earthquake was 10.96% [3].

Experimental AKI induced by glycerol injection is a well-established model of rhabdomyolysis [4]. It is characterised by intense cortical acute tubular necrosis and inflammatory cell infiltration [5,6]. Mesenchymal stem cells (MSCs) are one of the few stem cell types that has been applied as therapeutic agents for immunomodulation and tissue repair, which highlights their potential for restoration of kidney injury [7,8]. The mechanisms responsible for their protective roles most likely involve paracrine and endocrine effects, including mitogenic, anti-apoptotic and anti-inflammatory influences [9]. Several studies have shown that administration of exogenous MSCs contributes to the amelioration of glycerol-induced AKI [9-11]. However, its underlying mechanisms remain poorly understood.

Macrophages are key regulators of the innate immune system and are required for tissue homeostasis in regulation of the immune response and injury resolution [12]. It has been well documented that macrophages are critically involved in the initiation and orchestration of the acute inflammatory response and play an important role during injury and tubular cell apoptosis [13]. Inflammatory macrophages have been presumed to produce a wide array of pro-inflammatory mediators and induce apoptosis in target cells [14]. Depletion strategies have suggested that macrophages cause renal injury [15]. Current data suggest that macrophages perform both injury-inducing and repair-promoting tasks in different models of inflammation [16]. Several studies published within the past year demonstrate that macrophages or monocyte-derived dendritic cells, which were ablated during the repair phase of ischaemia/reperfusion injury in the kidney, resulted in deleterious outcomes [10,17-19].

The aim of this study was to explore the effects of MSCs on macrophage activation in rhabdomyolysis-induced AKI. Here, we show that administration of MSCs results in the amelioration of rhabdomyolysis-induced AKI and increased infiltration of M2 macrophages that are strongly associated with renal tubular cell proliferation. Depleting macrophages during the recovery phase attenuated the therapeutic effect of MSCs. Furthermore, we found that macrophages co-cultured with MSCs acquired an M2 phenotype and that administration of these anti-inflammatory macrophages into macrophage-depleted AKI mice also reduced kidney injury. MSCs can induce M2 macrophages, which mediate renoprotection of MSCs in rhabdomyolysis-induced AKI, both *in vivo* and *in vitro*.

## Methods

### Experimental animals and procedures

Eight- to twelve-week-old C57BL/6 male mice were obtained from the Experimental Animal Center of the Academy of Military Medical Sciences. The mice were housed at a constant room temperature with a 12-hour light/dark cycle. Standard rodent chow and water were provided *ad libitum*. The animals were acclimated for seven days prior to initiating the experiment. All animal protocols were approved by the Animal Ethics Committee of the Chinese PLA General Hospital and Military Medical College. Rhabdomyolysis (RM)-induced AKI was performed as previously described [10,11]. C57BL/6 mice were deprived of water for 24 hours and then administered half the dose of glycerol (50% v/v in sterile saline) in each hindlimb muscle under light sedation with pentobarbital. Dose-dependent studies defined an optimal dose of 8 mL/kg body weight of glycerol. Six hours later, the mice received an intravenous injection of MSCs (10^6^ in 200 μL normal saline) or an equal volume of normal saline (NS) into the tail vein.

The following groups were evaluated: Sham + NS, control plus normal saline injected after intramuscular injection of normal saline; Sham + MSCs, control plus MSC injected after intramuscular injection of normal saline; RM + NS, rhabdomyolysis plus normal saline injected after glycerol administration; and RM + MSCs, rhabdomyolysis plus 10^6^ MSC injected after glycerol administration. Each experimental group comprised 10 mice. At various time points after rhabdomyolysis, blood, kidney, lung and muscle samples were harvested for further processing.

*In vivo* depletion of macrophages was achieved by injecting an intravenous (iv) bolus of 100 μL/10 g body weight of clodronate liposomes (LC, FormuMax Scientific Inc, Palo Alto, California, USA) one day prior to rhabdomyolysis induction. Control mice were injected with plain control liposome vehicle (LV, FormuMax Scientific Inc, Palo Alto, California, USA).

### Cell culture

C57BL/6 mouse bone marrow–derived MSCs were obtained from Cyagen Biosciences (Cyagen Biosciences, Sunnyvale, CA, USA) and treated according to the manufacturer’s instructions. MSCs were placed in 25-cm^2^ culture flasks and cultured with MSC growth medium (Cyagen Biosciences) at 37°C under 5% CO_2_ and 90% humidity. The medium was changed every two days. The sixth to eighth passage MSCs were used for experiments.

RAW 264.7 cells, a murine macrophage-like cell line, were purchased from the Cell Bank of the Chinese Academy of Sciences (Shanghai, China). Macrophages were maintained in 25-cm^2^ culture flasks containing (Dulbecco’s) Modified Eagle’s Medium ((D)MEM) with high glucose and stable glutamine, supplemented with 100 units/mL penicillin, 100 μg/mL streptomycin, and 10% foetal calf serum (FCS) at 37°C under 5% CO_2_ and 90% humidity.

### Macrophage separation from the spleen and *ex vivo* co-culture with MSCs

C57BL/6 mice splenocytes were harvested and washed in ice-cold Roswell Park Memorial Institute (RPMI) 1640 medium (Gibco-BRL, Gaithersburg, MD, USA). The tissue was triturated using sterile syringes, and the resulting cell suspension was filtered via a 40-μm nylon mesh (BD Biosciences, North Ryde, Australia) and then incubated at 37°C for 30 minutes. The adherent cells were harvested and purified using MACS CD11b^+^ MicroBeads (Miltenyi Biotec, Bergisch Gladbach, Germany). These spleen-derived macrophages were rinsed three times with RPMI 1640 medium and cultured *ex vivo* with MSCs.

### Histopathological examination for acute tubular necrosis scores

The kidneys were fixed in 10% formalin for 24 hours and underwent routine dehydration and paraffin embedding. Renal tissues were sectioned at 3-μm thickness and stained with haematoxylin and eosin (H & E) and periodic acid–Schiff (PAS) using standard methods. Histological examinations were performed in a blind fashion. Acute tubular necrosis severity was quantified by counting the percentage of tubules that displayed cell necrosis, loss of brush border, cast formation and tubule dilatation as follows: 0 = none, 1 = ≤10%; 2 = 11% to 25%; 3 = 26% to 45%; 4 = 46% to 75%; and 5 = >76% [20]. Approximately 80 high-power fields (HPFs, ×400) per individual mouse (20 HPFs per slide, four slides per animal) were evaluated (n = five per group).

### Immunofluorescence staining

Mice were sacrificed at 24, 48 and 72 hours after rhabdomyolysis. Specimens were snap-frozen in optimum cutting temperature (O.C.T.) compound (Sakura Finetek USA, Torrance, CA, USA) and stored at – 80°C until further use. Frozen tissues were sectioned at 4-μm thickness and placed on poly-L-lysine pre-coated slides [21]. Sections were fixed in acetone at – 20°C for 10 minutes, washed three times with PBS and then blocked with 1% bovine serum albumin (BSA) for 30 minutes at room temperature. Primary antibodies for anti-F4/80 (1/100, clone BM8, eBioscience, San Diego, CA) and CD206 (1/100, MR5D3, Santa Cruz Biotechnology, Santa Cruz, CA, USA) were added, and the tissue was incubated overnight at 4°C. After washing in PBS three times, the sections were incubated with secondary antibodies conjugated with fluorescein isothiocyanate (FITC) or cyanin 3 (Cy3) (1/1000, Jackson ImmunoResearch Laboratories, West Grove, PA, USA) for one hour at room temperature in a darkened humidified chamber. Negative controls were not incubated with a primary antibody. Finally, the preparations were washed with PBS and mounted with fluorescent mounting medium containing 4',6-diamidino-2-phenylindole (DAPI) (Zhongshan Goldenbridge Biotechnology, Beijing, China). Each section was observed under a confocal laser scanning microscope (Olympus FluoView 1000, Tokyo, Japan) at a magnification of × 600.

RAW 264.7 macrophages were seeded onto six-well plates that were pre-treated with collagen I (Sigma-Aldrich, St. Louis, MO, USA) [22]. Each group was subjected to its own designated treatment regimen. The cells were fixed with 4% paraformaldehyde for five minutes at room temperature, followed by incubation at 4°C for ten minutes. Next, 1% BSA/PBS was sealed for 30 minutes at room temperature. The primary anti-F4/80 antibody (1/150, clone BM8, eBioscience) and CD206 antibody (1/150, MR5D3, Santa Cruz Biotechnology) were added to the cells and incubated overnight at 4°C. A secondary anti-rabbit antibody conjugated with FITC or Cy3 (1/1000, Jackson ImmunoResearch Laboratories) was incubated with the cells in the dark for one hour at room temperature. Finally, a fluorescent sealing liquid (Zhongshan Goldenbridge Biotechnology, Beijing, China) containing DAPI was added, and confocal laser scanning microscopy (Olympus FluoView 1000, Tokyo, Japan) was performed to determine the expression level of the macrophage surface antigens F4/80 and CD206, which are specific surface antigens for M2 macrophages.

### Immunohistochemistry

Immunohistochemical staining for the detection of proliferation of tubular cells was performed on formaldehyde-fixed and paraffin-embedded tissues using the avidin-biotin immunoperoxidase method [23]. Kidney sections were subjected to antigen retrieval, and sections were blocked and labelled overnight at 4°C with rabbit anti-proliferating cell nuclear antigen (PCNA) (1/100, Santa Cruz Biotechnology). After incubation with horseradish peroxidase (HRP)-conjugated secondary antibodies (Dako, Glostrup, Denmark), the sections were treated with avidin-biotin peroxidase conjugate (ABC Kit, Vector Laboratories, Burlingame, CA, USA). The reaction was visualised using a 3,3′-diaminobenzidine (DAB) chromogen (Dako) following tissue counterstaining with haematoxylin.

### Western blotting analysis

Mouse kidney homogenates (100 μg of total protein) were separated on polyacrylamide-SDS gel and electroblotted onto nitrocellulose membrane. After blocking with 5% non-fat dry milk, the membrane was incubated with antibodies against mouse CD206 (1/1000, MR5D3, Santa Cruz Biotechnology) at 4°C overnight, followed by incubation with a HRP-conjugated secondary antibody. β-actin (Santa Cruz Biotechnology) was used as the loading control, and immunoreactive bands were visualised using enhanced chemiluminescence.

### Enzyme-linked immunosorbent assay of cytokines

The concentrations of IL-6, IL-10 and TNF-α in the mouse serum and supernatants of co-cultured macrophages were detected using enzyme-linked immunosorbnt assay (ELISA) kits (R&D Systems, Minneapolis, MN, USA). The optical density (OD) was measured at 450 nm using a microplate reader (Thermo Fisher Scientific, Waltham, MA, USA) with the correction wavelength set at 540 nm.

### Real-time PCR

Total RNA was isolated using TRIzol (Invitrogen, Carlsbad, CA, USA). The TaqMan Reverse Transcription Kit (Applied Biosystems, Foster City, CA, USA) and a Gene Amp® PCR System 9700 (Applied Biosystems) were used to generate cDNA. Gene expression analysis was determined by quantitative real-time PCR using the SYBR Green Mastermix and a 7500 Real-time PCR System (Applied Biosystems). The results were analysed using the 2^-ΔΔCT^ method with normalisation against glyceraldehyde 3-phosphate dehydrogenase (GAPDH) expression (n = 5 for each group). The following primers were used: TNF-α Fw: AGGCACTCCCCCAAAAGATG, Rev: CTTGGTGGTTTGCTACGACG; inducible nitric oxide synthase (iNOS) Fw: CAGA- TCGAGCCCTGGAAGAC, Rev: CTGGTCCATGCAGACAACCT; IL-10 Fw: AGGCAGC- CTTGCAGAAAAGA, Rev: GCTCCACTGCCTTGCTCTTA; mannose receptor (MR) Fw: CAAGGAAGGT- TGG-CATTTGT, Rev: CCTTTCAGTCCTTTGCAAGC; and GADPH Fw: TATTGGGC- GCCTGG- TCACCA, Rev: TTAGTGGGGTCTCGCTCCT GGAAG.

### *In vivo* tracking of MSCs

Red Fluorescence Protein (RFP)-labelled MSCs (10^6^) were injected into mice six hours after rhabdomyolysis via the tail vein. *In vivo* tracking of MSCs in muscle, lung and kidney was performed using a Leica two-photon fluorescence confocal imaging TCS SP5 system (Leica Microsystems, Mannheim, Germany) after rhabdomyolysis. Simultaneously, samples were also harvested and snap-frozen with O.C.T compound (Sakura Finetek) to follow the redistribution of MSCs. Slides were fixed in acetone at – 20°C for 10 minutes and stained with mounting medium containing DAPI (Zhongshan Goldenbridge Biotechnology). All trackings were observed at a magnification of × 600.

### *Ex vivo* culture conditions for macrophages and co-culture protocol

RAW 264.7 macrophages were divided into three groups according to treatment: macrophages cultured in normal medium for 48 hours were defined as M0; macrophages undergoing a 2 hour incubation with lipopolysaccharide (LPS, Sigma-Aldrich, Castle Hill, Australia) (2.5 mg/mL) and then cultured in normal medium for another 46 hours were defined as M1 [24]; and macrophages co-cultured with MSCs for 72 hours were defined as M2. For transwell co-cultures, a 0.4-μm pore size insert (Corning, Lowell, MA, USA) was placed into the six-well plate with LPS-stimulated macrophages on the bottom well, while 2 × 10^5^ MSCs were seeded onto inserts and cultured for another 72 hours [23].

### Flow cytometric analysis

For surface staining, macrophages were harvested using a cell scraper, and Fc receptors were blocked with an Fc receptor blocking agent (Miltenyi Biotech) for 15 minutes at 4°C. After staining for CD206: FITC (MR5D3, AbD), the cells were processed with the BD Cytofix/Cytoper Fixation/Permeabilisation kit (BD Biosciences), followed by incubation with IL-10 (JES5-16E3, eBiosciences) and analysis using flow cytometry (FC500 MPL, Beckman Coulter, Brea, CA, USA).

### Statistical analysis

Statistical analysis was performed using the IBM SPSS Statistics 17.0.2 software (IBM Corporation, Armonk, NY, USA). Results are presented as the mean values ± standard deviation (SD). Multiple comparisons of parametric data were performed using one-way analysis of variance (ANOVA) followed by Student–Newman–Keuls *post-hoc* tests. The Student*’*s *t*-test was used to compare differences between means. A *P*-value of <0.05 was considered significant.

## Results

### MSCs protect against glycerol-induced AKI

**Figure 1:**
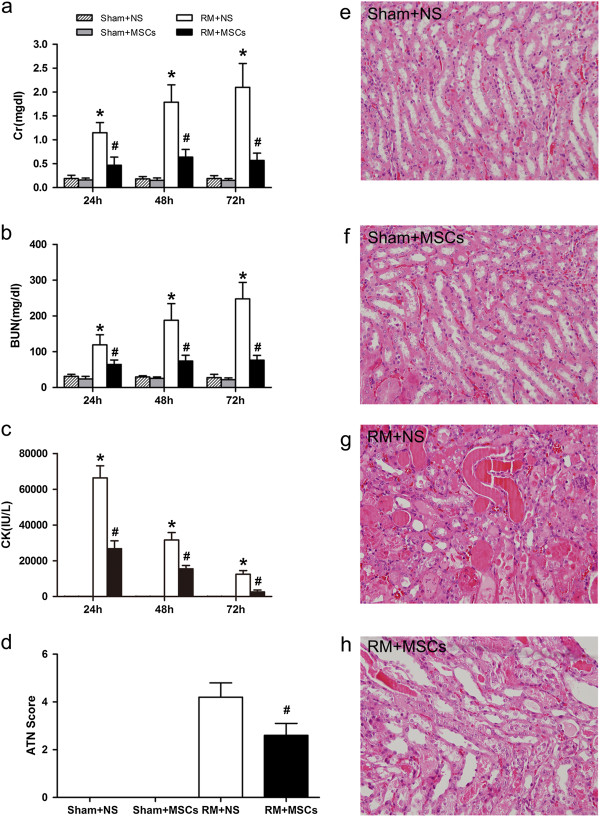
**Mesenchymal stem cells (MSCs) ameliorate rhabdomyolysis (RM)-induced acute kidney injury (AKI). (a-c)** Compared with animals treated with saline, infused MSCs significantly reduced serum creatinine (SCr **(a)**), blood urea nitrogen (BUN **(b)**) and serum phosphocreatine kinase (CK**(c)**) levels 24, 48 and 72 hours after RM, and there were no significant changes following treatment of sham mice. *P <0.05 versus sham, ^#^*P* <0.05 versus rhabdomyolysis treated with saline at the corresponding times (n = 10). **(d)** MSC therapy markedly reduced acute tubular necrosis (ATN) scores 72 hours after rhabdomyolysis. **(e-h)** Treatment with the sham-operated control (Sham + NS and Sham + MSCs) had no effect on renal histopathological parameters **(e and f)**, while MSC therapy markedly improved tubular injury **(h)**, and mice treated with saline showed severe tubular injury **(g)** (HPFs, ×400, n = 10). HPFs, high-power fields; NS, normal saline.

To determine whether MSCs attenuate rhabdomyolysis-induced AKI, we subjected the mice to an intramuscular injection with 50% glycerol solution following water deprivation for 24 hours. We administered MSCs (10^6^ per mouse) six hours after rhabdomyolysis. MSC infusion markedly reduced the levels of serum creatinine (SCr, Figure 1a), blood urea nitrogen (BUN, Figure 1b) and phosphocreatine kinase (CK, Figure 1c) 24, 48 and 72 hours after glycerol injection compared with mice administered saline. Because the maximum AKI was achieved at 72 hours in our study, we selected the 72-hour time point to evaluate kidney injury. Sham mice with an intramuscular injection of NS (Figure 1e) or MSCs (Figure 1f) did not show any significant tubular damage. Rhabdomyolysis (RM) mice receiving normal saline treatment showed tubular necrosis, tubular dilatation and cast formation (Figure 1g). Infused MSCs markedly improved tubular injury (Figure 1h) and lowered the AKI score (Figure 1d) after 72 hours of rhabdomyolysis.

### Distribution of MSCs and alleviation of tissue damage and inflammation

**Figure 2:**
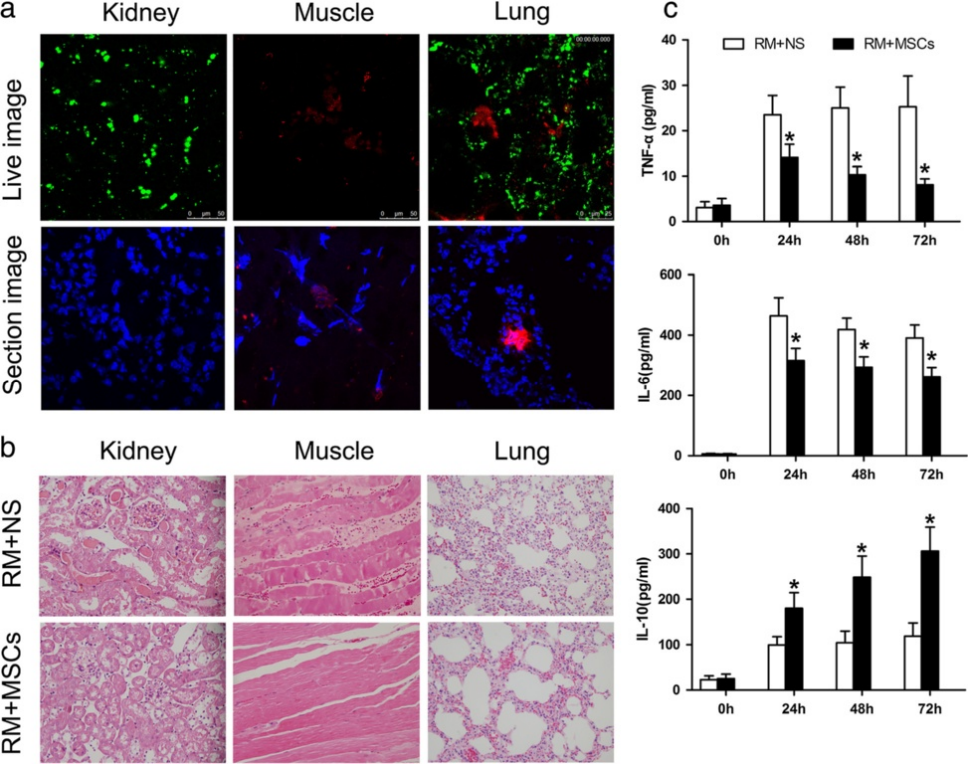
**Infused MSCs persist in the lung and muscle and suppress tissue damage. (a)** MSCs persisted in the lung and injured muscle 24 hours after rhabdomyolysis. RFP-labelled MSCs (red) were detected *in vivo* using two-photon fluorescence confocal microscopy. Nuclei were stained with DAPI (blue). Original magnification, ×600, n = 3. **(b)** Representative H & E-stained paraffin-embedded sections of kidney, gastrocnemius muscle and lung with or without administration of MSCs (n = 10). Mice were sacrificed 24 hours after rhabdomyolysis. **(c)** ELISA showed that MSC treatment significantly decreased the levels of both of the pro-inflammatory cytokines TNF-α and IL-6 and increased the anti-inflammatory cytokine IL-10. *P <0.05 versus RM + NS, n = 5. DAPI, 4,6-diamidino-2-phenylindole; MSCs, mesenchymal stem cells; NS, normal saline; RM, rhabdomyolysis.

To evaluate the distribution of MSCs after AKI, RFP-labelled MSCs were infused into mice six hours after rhabdomyolysis. *In vivo* imaging was performed 24 hours after rhabdomyolysis using two-photon fluorescence confocal microscopy. The distribution was also confirmed by corresponding frozen sections. Twenty-four hours after rhabdomyolysis, signals were detected in the lung and gastrocnemius muscle. However, no apparent MSCs were observed in the injured kidney (Figure 2a). Rhabdomyolysis caused multiple organ damage, including significant oedema in the gastrocnemius muscles with degeneration and atrophy in muscle fibres, prominent intra-alveolar and interstitial oedema with hyaline membrane formation, as well as acute tubular necrosis. Our results showed that mice receiving MSCs displayed decreased tissue damage in the kidney, muscle and lung, compared with control mice receiving normal saline injection (Figure 2b). In addition, an ELISA showed that MSC treatment significantly decreased serum levels of the pro-inflammatory cytokines tumour necrosis factor alpha (TNF-α) and interleukin 6 (IL-6) and increased the anti-inflammatory cytokine IL-10 (Figure 2c). These findings suggest that administration of MSCs exerted a therapeutic effect and ameliorated the progression of organ damage via unique immunomodulatory functions that inhibit the pro-inflammatory progression of injury and elicit an anti-inflammatory effect.

### MSCs accelerate M2-polarised macrophage infiltration and induce tubular cell proliferation

**Figure 3:**
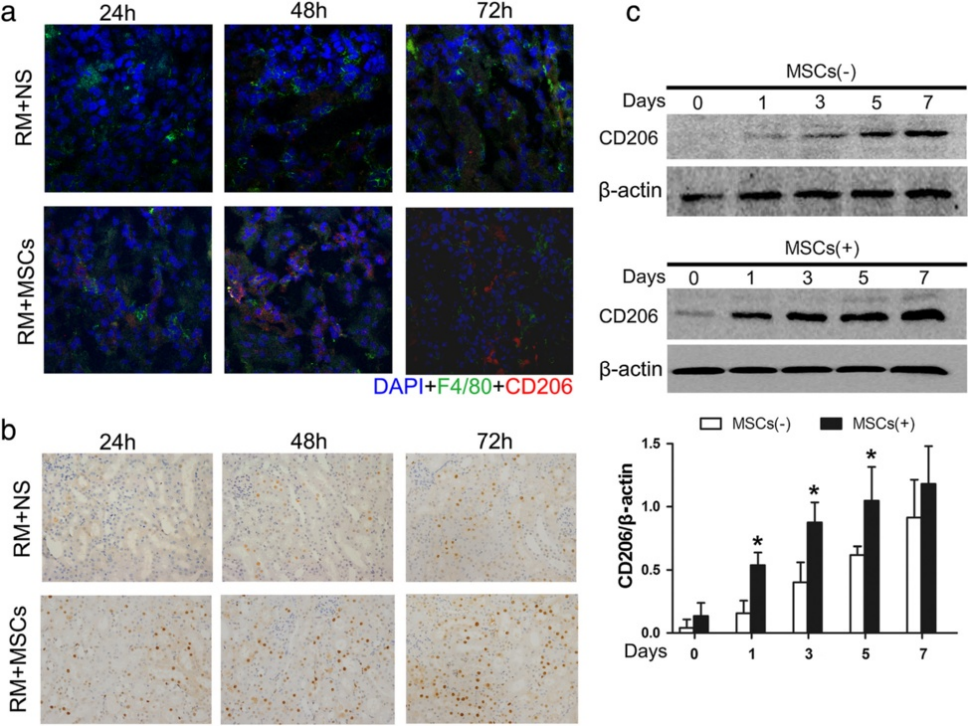
**MSCs increase infiltration of M2-polarised macrophages in rhabdomyolysis kidney and promote tubular cell regeneration. (a)** Representative confocal microscopy images of macrophages (F4/80) and M2-polarised macrophage (CD206) infiltration in kidneys after saline or MSC infusion. **(b)** PCNA–positive nuclei within the tubuli of glycerol-treated mice treated with saline or MSCs at various time intervals (24, 48 and 72 hours). **(c)** Representative western blotting analysis showing time-dependent increases in mannose receptor (CD206) expression induced by MSC treatment. *P <0.05 versus RM + NS, n = 3. MSC, mesenchymal stem cell; NS, normal saline; PCNA, proliferating cell nuclear antigen; RM, rhabdomyolysis.

Representative confocal micrographs of kidneys showed the presence of macrophages and M2-polarised macrophages in RM + NS and RM + MSCs groups after 24, 48 and 72 hours of rhabdomyolysis (Figure 3a). Macrophages were detected using green fluorescence with an antibody specific for the mouse macrophage marker F4/80, and M2-polarised macrophages were detected using red fluorescence with an antibody specific for the CD206 marker. Nuclei were counterstained with DAPI (blue). Macrophage infiltration increased after rhabdomyolysis, and treatment with MSCs accelerated M2-polarised macrophage infiltration, as represented by immunostaining. Western blotting analysis demonstrated a time-dependent increase in CD206 protein expression in the kidney after rhabdomyolysis (Figure 3c). This observation suggested that macrophage presentation in MSC treatment had adopted a phenotype similar to that of alternatively-activated macrophages. As shown in Figure 3b, MSCs also significantly enhanced tubular cell proliferation compared with saline treatment alone, as detected by PCNA-positive cells after 24, 48 and 72 hours of rhabdomyolysis. These compelling findings suggested that MSCs were capable of promoting infiltration of alternatively-activated macrophages in the wounded kidney, which potentially contributed to the regulation of the inflammatory response and enhanced the healing of rhabdomyolysis.

### Depletion of macrophages delays the recovery of MSC-treated AKI

**Figure 4:**
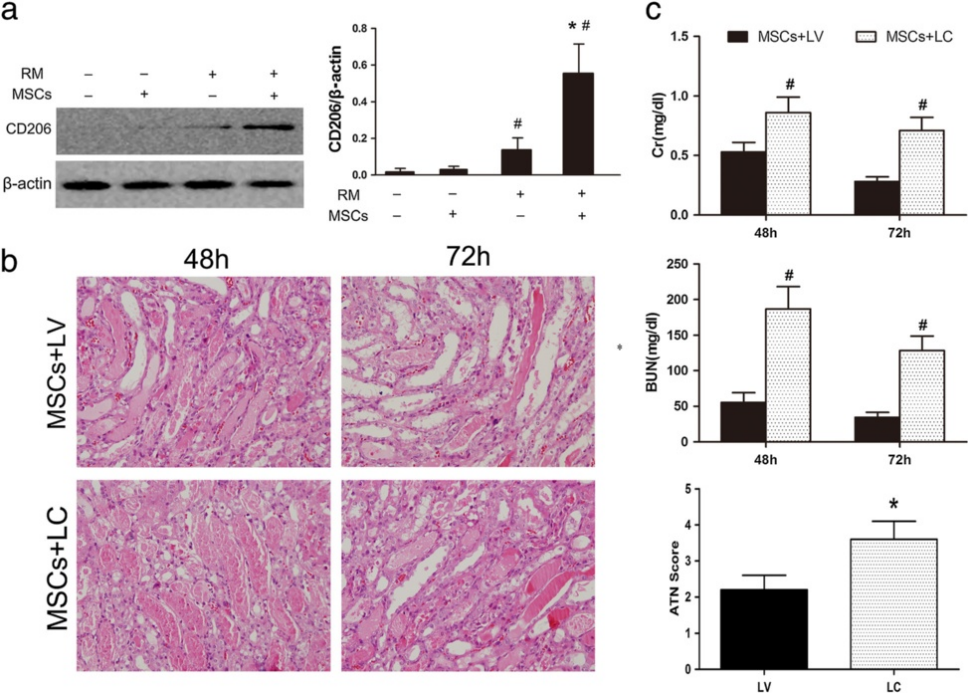
**MSCs induce M2 macrophages 24 hours after rhabdomyolysis, and macrophage depletion delays recovery of AKI. (a)** Western blotting analysis of mannose receptor (CD206) expression in the kidney 24 hours after rhabdomyolysis. ^#^*P* <0.05 versus RM(−), *P <0.01 versus RM + NS, n = 3. **(b)** Renal histopathology observed after 48 and 72 hours of MSC-treated rhabdomyolysis in mice for the liposomal vehicle (LV) control group (MSCs + LV) and liposomal-encapsulated clodronate (LC) group (MSCs + LC) (HPFs, ×400, n = 5). **(c)** Serum creatinine (SCr), blood urea nitrogen (BUN) and acute tubular necrosis (ATN) scores of MSC-treated rhabdomyolysis mice treated with LC or LV. *P <0.05 versus MSCs + LV, ^#^*P* <0.01 versus MSCs + LV, n = 5. AKI, acute kidney injury; HPFs, high-power fields; MSCs, mesenchymal stem cells; NS, normal saline; RM, rhabdomyolysis.

**Figure 5:**
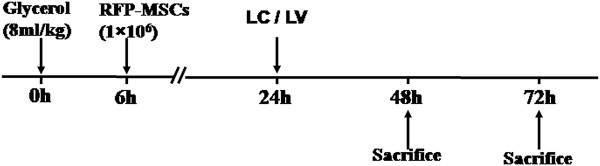
**Experimental schedule of macrophage depletion 24 hours after rhabdomyolysis.** A bolus of 100 μL/10 g body weight of liposomal-encapsulated clodronate (LC) was injected into MSC-treated AKI mice to deplete M2-polarised macrophages, and a liposomal vehicle (LV) was administered as a control. AKI, acute kidney injury; MSC, mesenchymal stem cells.

It was determined that CD206^+^, non-inflammatory (M2) macrophages predominate in the RM + MSCs group. Twenty-four hours after rhabdomyolysis, western blotting analysis revealed that RM mice treated with MSCs showed strong expression of CD206 in the kidney, while RM mice infused with normal saline showed weak expression. Independent of receiving normal saline or MSC treatment, the sham control did not show any CD206 expression (Figure 4a). In addition, at 24 hours, a bolus of 100 μL/10 g body weight of LC was injected into MSC-treated AKI mice to deplete M2-polarised macrophages, and a LV was administered as a control (Figure 5). Renal histopathology and function analyses showed that depletion of M2-polarised macrophages can resume tubular injury at 72 hours (Figure 4b, 4c). In MSC-treated mice, animals injected with LC compared with LV, still showed higher levels of SCr and BUN and aggravation of tubular injury 72 hours after rhabdomyolysis.

### Effect of co-culture with MSCs on mRNA expression

**Figure 6:**
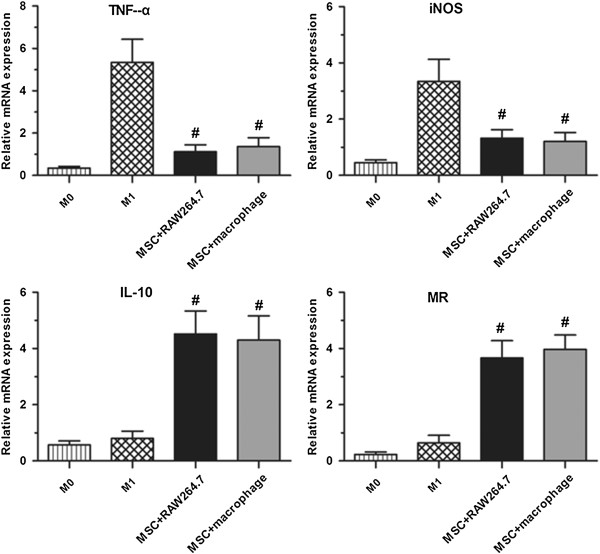
**Effect of co-culture with MSCs on mRNA expression. **MSCs reduce macrophage gene transcriptional activity of TNF-a and iNOS and promote gene transcriptional activity of IL-10 and MR in the RAW264.7 cell line and macrophages of the C57BL/6 strain. ^#^*P* <0.01 versus M0 and M1. iNOS, inducible nitric oxide synthase; MSCs, mesenchymal stem cells.

To investigate whether RAW264.7 cells have similar immunogenicity as macrophages of the C57BL/6 strain, we examined the level of TNF-α, iNOS, IL-10 and MR using RT-PCR. Our results showed that compared with the M0 and M1 groups, the mRNA expression levels of TNF-α and iNOS were significantly decreased and the mRNA expression levels of IL-10 and MR were significantly increased in co-culture groups of RAW264.7 cells and macrophages of the C57BL/6 strain at 24 hours (Figure 6).

### MSCs induce M2-polarised macrophages *in vitro*

**Figure 7:**
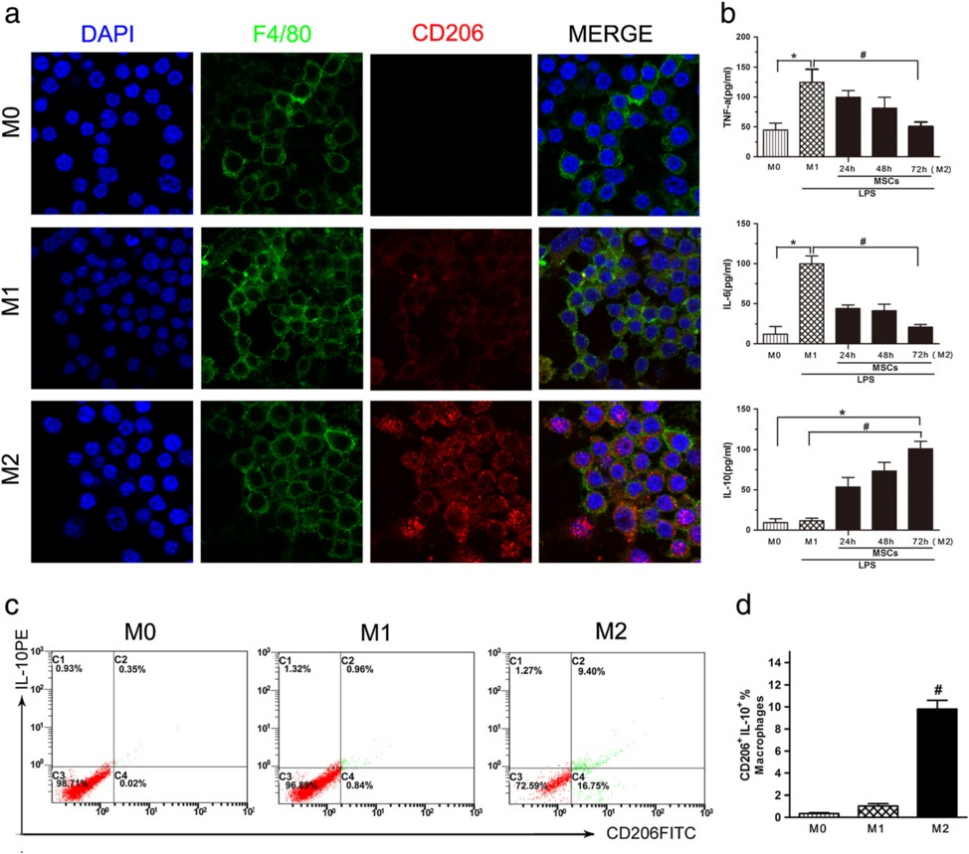
**Comparison of cell surface markers and inflammatory factor expression in M0, M1 and M2 macrophages. (a)** Cell surface marker staining of F4/80 and CD206 expression in M0, M1 and M2 macrophages. RAW264.7 macrophages cultured in normal medium were defined as M0 with significant F4/80 and negative CD206 expression. RAW264.7 macrophages stimulated with LPS were defined as M1 with significant F4/80 and weak CD206 expression. RAW264.7 macrophages stimulated with LPS and co-cultured with MSCs in transwells were defined as M2 with significant expression of F4/80 and CD206. **(b)** ELISA showed that MSC treatment significantly decreased the levels of the pro-inflammatory cytokines TNF-α and IL-6 and increased the levels of the anti-inflammatory cytokine IL-10 in culture medium. *P <0.05 versus M0, ^#^*P* <0.05 versus M2, n = 3. **(c-d)** MSCs increased the percentage of CD206^+^ IL-10^+^ cells in RAW264.7 macrophages after co-culture for 72 hours. ^#^*P* <0.05 versus M0 or M1, n = 3. MSCs, mesenchymal stem cells.

Next, we investigated the hypothesis that the interaction of MSCs with macrophages plays a significant role in their anti-inflammatory and immune modulatory effects. RAW 264.7 cells, a murine macrophage-like cell line, were cultured in normal medium and defined as M0, and M0 stimulated with LPS was defined as M1. M1 co-cultured with MSCs for 72 hours with significantly increased expression of CD206, as assessed using polarised immunofluorescence staining, was defined as M2 (Figure 7a). To determine whether secreted factors were responsible for the transition of the macrophage phenotype, transwell inserts were used during co-culture to prevent direct physical contact between the MSCs and macrophages. As MSC-co-cultured macrophages expressed the anti-inflammatory macrophage marker CD206, intracellular cytokine staining utilising established stimulation protocols [25,26] was performed to further characterise their immunophenotype. Flow cytometry analysis showed that MSC-co-cultured M2 macrophages produced more IL-10 (Figure 7c and d) compared to M0 and M1 macrophages. In addition, ELISA analysis showed that after co-culture with MSCs, the levels of the pro-inflammatory cytokines TNF-a and IL-6 in the medium of RAW 264.7 cells were significantly decreased, while that of the anti-inflammatory cytokine IL-10 was accordingly increased (Figure 7b).

### MSC–educated macrophages improve kidney injury and promote kidney repair

**Figure 8:**
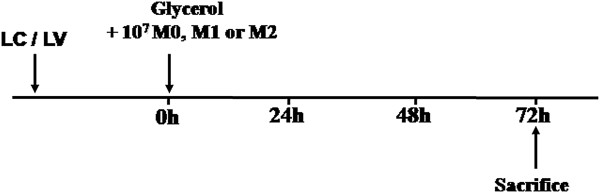
**Experimental schedule of macrophage adoptive transfer.** Intravenous (iv) bolus of 100 μL/10 g body weight of clodronate liposomes (LC) was used to deplete macrophages one day prior to rhabdomyolysis induction. Control mice were injected with plain control liposomes (LV). Three different phenotypes of macrophages were infused into mice with rhabdomyolysis.

**Figure 9:**
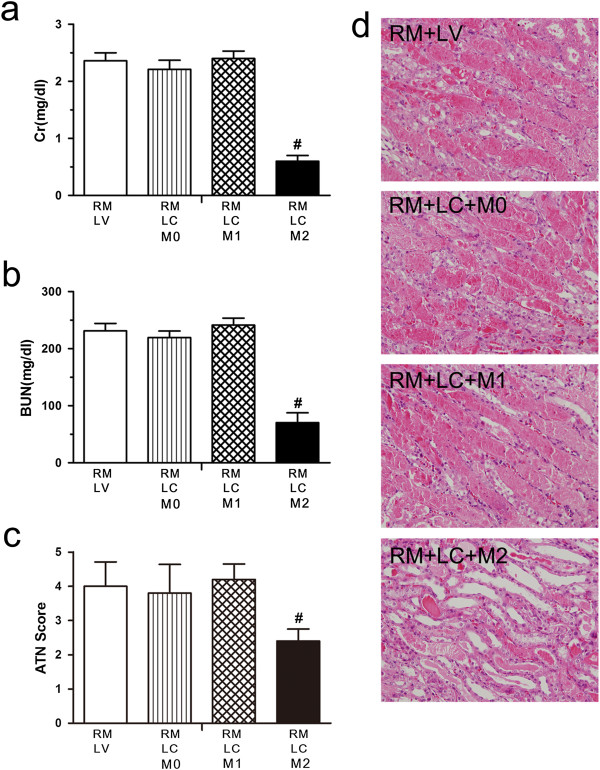
**MSC–educated M2 macrophages improve kidney injury and promote kidney repair.** Mice treated with LV or LC were subjected to rhabdomyolysis followed by infusion of 1 × 10^7^ M0, M1 or M2 macrophages immediately after glycerol injection. **(a-c)** Serum creatinine (SCr) values, blood urea nitrogen (BUN) values and acute tubular necrosis (ATN) scores are shown for mice 72 hours after rhabdomyolysis. ^#^*P <*0.05 versus rhabdomyolysis LV alone, M0 or M1 groups, n = 5. **(d)** Representative renal histopathology of different groups is shown 72 hours after rhabdomyolysis. LC, liposomal clodronate; LV, liposomal vehicle; MSC, mesenchymal stem cell.

To determine whether MSC-induced macrophages exert different effects following kidney injury, circulating and tissue myeloid phagocytes, including monocytes and resident macrophages, were depleted by LC. Mice with LV infusion were treated as a negative control. After two days, all mice received an intramuscular injection of glycerol to induce rhabdomyolysis. Next, 1 × 10^7^ pre-treated macrophages were injected into macrophage-depleted mice (Figure 8). Animals infused with M0 macrophages or M1 macrophages demonstrated increased levels of SCr and BUN that were indistinguishable from LV-treated control animals 72 hours after injury. Simultaneously, M2 macrophages also ameliorated rhabdomyolysis-induced renal injury (Figure 9a, 9b). Examination of renal histology (Figure 9d) and tubular injury scoring (Figure 9c) 72 hours after rhabdomyolysis confirmed that tubule damage was less severe in M2 macrophage-treated animals compared with LV alone or M0 and M1 macrophage-infused mice. Thus, these data demonstrated that MSCs ameliorate rhabdomyolysis-induced AKI via eliciting the polarisation of M2 macrophages.

## Discussion

In the present study, we demonstrated several findings regarding the mechanism of MSC therapy in rhabdomyolysis-induced AKI: (1) MSCs increased the percentage of macrophages and the expression of M2-polarised macrophages in the injured kidney, with morphological and functional recovery and proliferation of renal tubular epithelial cells; (2) MSC-conferred renoprotection is not entirely attributed to their ability to home and engraft to the site of kidney damage but may act via their unique abilities to modulate the inflammatory immune response and transition from a pro-inflammatory profile to an anti-inflammatory profile; (3) MSC-mediated renoprotection was blunted by the depletion of macrophages in the repair period; (4) RAW 264.7 cells co-cultured with MSCs not only acquired an anti-inflammatory M2 phenotype but also secreted trophic factors, such as IL-10; and (5) adoptive transfer of MSC–educated macrophages improved kidney injury and promoted kidney repair. Our data suggest that MSCs can promote a subsequent switch to an alternatively-activated macrophage phenotype, resulting in the suppression of the inflammatory response and promotion of a proliferative repair phase in rhabdomyolysis-induced AKI, which might be one of the potential mechanisms underlying MSC therapy in AKI.

Experimental evidence suggests that intra-renal vasoconstriction, direct and ischaemic tubule injury, and tubular obstruction all play a role in the development of rhabdomyolysis-induced AKI [27]. Vascular mediators in the reduction of renal blood flow appear to be locally stimulated by inflammation as a result of endothelial dysfunction, which is common to other forms of AKI [28]. The ability of MSCs to elicit repair via paracrine and/or endocrine mechanisms, such as to inhibit the release of pro-inflammatory cytokines and to secrete a variety of trophic growth factors, may collectively mediate the protective and regenerative effects of AKI in laboratory rodents [11,23,29-32]. Here, the *in vivo* tracking of fluorescently labelled MSCs demonstrated that these cells did not appear in the site of kidney injury, which indicated that the mechanisms in which MSCs confer protection are not entirely attributed to their ability to home and engraft to the site of kidney damage. We found that administration of MSCs following glycerol injection resulted in a significant decrease in the levels of serum pro-inflammatory cytokines, such as IL-6 and TNF-α, and increased levels of the anti-inflammatory cytokine IL-10, which caused accelerated tubular epithelial cell proliferation and improved renal function 24, 48 and 72 hours after rhabdomyolysis compared with saline-treated mice. These beneficial effects were also correlated with increased infiltration of macrophages, suggesting that immunomodulation, particularly via the activation of macrophages, may play an important role in MSC therapy in AKI.

Do MSCs act via macrophages? Despite current data showing therapeutic efficacy, the precise manner in which MSCs confer renoprotection is not completely understood. Macrophages play a crucial role in innate and adaptive immunity and are believed to exist in one of two opposing polarisation states. Classically-activated or M1 macrophages display a pro-inflammatory profile, whereas alternatively-activated or M2 macrophages express anti-inflammatory and tissue repair properties [33]. M2 macrophages appear to be major players in the immune system by preventing excessive inflammation while downregulating host protection against various pathogens. These effects are linked to the modulation of the expression of receptors at the cell surface and by modification of endocytic and phagocytic pathways. It has been reported that exposure to IL-4 and IL-13 can induce an M2 alternative polarisation state [34]. One characteristic of M2 macrophages is the increase in MR (CD206) expression [35]. In our study, compared with saline infusion, MSC treatment accelerated M2-polarised macrophage infiltration, as shown by immunofluorescence using specific antibodies for CD206, and it induced a comparable enhancement of tubular cell proliferation. Western blotting analysis also demonstrated a time-dependent increase in CD206 protein expression. These observations suggest that macrophages present in MSC treatment adopted a phenotype similar to that of alternatively-activated macrophages and resulted in renoprotection.

Is macrophage induction important in MSC therapy? Inflammatory cues within the regional microenvironment can prime the macrophage phenotype and determine whether these cells will have a beneficial or deleterious effect during tissue repair and remodelling. MSCs have been found to act via their unique immunomodulatory abilities, which can alter the pro-inflammatory course of injury. Thus, they might serve as guardians against excessive inflammatory responses in various modes, such as the *in vitro* conversion of macrophages to the IL-10-secreting phenotype [24]. Subsequently, the macrophage phenotype and function may ultimately determine the outcome of inflammation [34]. Macrophage depletion, via administration of LC, has been reported to diminish experimental AKI by abrogating persistent inflammation and subsequent development of fibrosis [19]. Our study showed that in the first 24 hours of rhabdomyolysis, MSC treatment elevated the expression of CD206^+^ M2 macrophages. However, saline-treated mice showed weak expression. When LC was administered to deplete macrophages, the attenuation of MSCs in renal injury was abrogated. Taken together, these findings further indicate the critical role of M2 macrophage induction in MSC therapy in AKI of rhabdomyolysis.

Can MSCs modulate the state of macrophage activation and directly affect the outcome of AKI? Recent studies have shown that *in vitro* co-culture of human MSCs and macrophages resulted in an alternatively-activated macrophage phenotype described as MR^high^ and IL-10^high^ with enhanced phagocytic activity [24,36]. These results provide *in vitro* evidence and confirm the belief that the activation state governing macrophage function is dependent on the inflammatory stimuli received from the microenvironment. Using an adoptive transfer model, we found that amelioration of AKI was dependent on the transfer of macrophages co-cultured with MSCs after glycerol injection. Examination of renal histology and tubular injury scoring confirmed that tubule damage was less severe in M2 macrophage-treated animals compared to the unactivated macrophages and LPS-stimulated macrophage groups. Collectively, these data suggested that the modulation state of M2 macrophages was the underlying mechanism in MSC-mediated protection in rhabdomyolysis-induced AKI. Our co-culture assays using transwell membranes showed that the interaction of MSCs and macrophages was not due to cell-to-cell contact but more likely to the production of MSC-derived trophic factors. Importantly, we should emphasise that many of these results, while suggestive of the role of M2, do not provide direct evidence for it. Mechanisms underlying macrophage polarisation to a trophic phenotype and promotion of tubular repair have yet to be fully elucidated in MSC therapy.

We think that our study of the roles of M2 macrophages in the mechanism of MSC therapy in rhabdomyolysis-induced AKI should provide the basis for the future functional studies needed to define the efficacy of MSC more accurately. While results from animal models have shown great potential of MSC, experimental data does have limitations. For example, the findings of a murine study may not necessarily apply to humans, and there are differences between human and mouse MSCs and macrophages. Further studies in clinical applications to dissect the role of MSC in AKI would be desirable.

## Conclusions

Our findings suggest that MSCs can ameliorate rhabdomyolysis-induced AKI via the activation of macrophages to a trophic M2 phenotype, which supports the transition from tubule injury to tubule repair. Our study also offers additional evidence that administered MSCs protect against AKI and accelerate the recovery phase in a rodent model of rhabdomyolysis and sheds new light on the mechanisms of the beneficial effects of MSCs on AKI.

## Abbreviations

AKI: acute kidney injury; BSA: bovine serum albumin; BUN: blood urea nitrogen; CK: phosphocreatine kinase; Cy3: cyanine 3; DAB: 3,3′-diaminobenzidine; DAPI: 4,6-diamidino-2-phenylindole; ELISA: enzyme-linked immunosorbent assay; FCS: foetal calf serum; FITC: fluorescein isothiocyanate; H & E: haematoxylin and eosin; HPFs: high-power fields; IL-10: interleukin-10; IL-6: interleukin-6; iNOS: inducible nitric oxide synthase; LC: liposomal clodronate; LPS: lipopolysaccharide; LV: liposomal vehicle; MR: mannose receptor; MSC: mesenchymal stem cell; NS: normal saline; O.C.T.: optimum cutting temperature; PAS: periodic acid–Schiff; PBS: phosphate-buffered saline; PCNA: proliferating cell nuclear antigen; RM: rhabdomyolysis; SCr: serum creatinine; TNF-α: tumour necrosis factor alpha.

## Competing interests

The authors declare that they have no competing interests.

## Authors’ contributions

YQG conceived and designed the experiments, performed the experiments, analyzed the data, and wrote the paper. LZ and BF performed the experiments, analyzed the data, and wrote the paper. JRZ, QH, JH, DGL and CJL conceived and performed the experiments and analyzed the data. SYC and FZ performed the experiments and analyzed the data. LZ and XMC conceived and revised the paper critically for important intellectual content, and gave final approval of the version to be published. All authors read and approved the final manuscript.
